# Endoplasmic Reticulum Redox State Is Not Perturbed by Pharmacological or Pathological Endoplasmic Reticulum Stress in Live Pancreatic β-Cells

**DOI:** 10.1371/journal.pone.0048626

**Published:** 2012-11-08

**Authors:** Irmgard Schuiki, Liling Zhang, Allen Volchuk

**Affiliations:** 1 Division of Cellular and Molecular Biology, Toronto General Research Institute, University Health Network, Toronto, Ontario, Canada; 2 Department of Biochemistry, University of Toronto, Toronto, Ontario, Canada; 3 Department of Physiology, University of Toronto, Toronto, Ontario, Canada; University of Pittsburgh, United States of America

## Abstract

Accumulation of unfolded, misfolded and aggregated proteins in the endoplasmic reticulum (ER) causes ER stress. ER stress can result from physiological situations such as acute increases in secretory protein biosynthesis or pathological conditions that perturb ER homeostasis such as alterations in the ER redox state. Here we monitored ER redox together with transcriptional output of the Unfolded Protein Response (UPR) in INS-1 insulinoma cells stably expressing eroGFP (ER-redox-sensor) and mCherry protein driven by a GRP78 promoter (UPR-sensor). Live cell imaging, flow cytometry and biochemical characterization were used to examine these parameters in response to various conditions known to induce ER stress. As expected, treatment of the cells with the reducing agent dithiothreitol caused a decrease in the oxidation state of the ER accompanied by an increase in XBP-1 splicing. Unexpectedly however, other treatments including tunicamycin, thapsigargin, DL-homocysteine, elevated free fatty acids or high glucose had essentially no influence on the ER redox state, despite inducing ER stress. Comparable results were obtained with dispersed rat islet cells expressing eroGFP. Thus, unlike in yeast cells, ER stress in pancreatic β-cells is not associated with a more reducing ER environment.

## Introduction

Biosynthesis, folding and maturation of membrane and secretory proteins occur in the ER. The ER lumen has a high calcium concentration and an oxidizing environment compared to the cytosol that are important for normal ER chaperone function and for proper folding of proteins that contain disulfide bonds [1]–[3]. Reducing agents disrupt the oxidizing ER environment critical for disulfide bond formation, which leads to accumulation of unfolded and misfolded proteins causing ER stress. In addition to altered ER redox, ER stress can result from a variety of physiological or pathological conditions such as loss of ER luminal calcium or inhibition of posttranslational modification of secretory proteins.

Cells respond to ER stress by activating the Unfolded Protein Response (UPR) that transiently inhibits translation to reduce the amount of new protein synthesis and enhances folding capacity by increasing ER chaperones and ER-associated degradation (ERAD) [4]–[6]. In mammalian cells, the UPR is activated by the coordinate action of three ER stress sensors that reside in the ER membrane, RNA-dependent protein kinase-like ER kinase (PERK), inositol-requiring enzyme-1 (IRE-1), and activating transcription factor 6 (ATF6) [5]–[7]. Because quantification of misfolded or unfolded proteins directly is not possible ER stress is typically monitored by evaluating whether the UPR sensors are activated or by examining the levels of UPR response genes such as chaperone proteins. However, this indirect way of measuring ER stress has limitations, such as in the case of disabled or non-functional UPR systems. Moreover, it is difficult to determine if activation of the UPR is actually successful in alleviating ER stress and re-establishing ER homeostasis. Recently, a redox sensitive GFP (eroGFP) was used in yeast to monitor the redox environment in the ER of living cells under control and ER stress conditions [8]. eroGFP is a variant of the *Aequorea victoria* green fluorescent protein (GFP) generated by substitution of surface-exposed residues with cysteines. Changes in the oxidation state of the introduced redox-reactive group (cystein pair) leads to reversible formation of an intramolecular disulfide bridge and spectral changes in the eroGFP's chromophore. Interestingly, chemical inducers of ER stress such as tunicamycin (Tm) altered the redox potential in the ER of yeast cells, which is normally oxidizing, to a more reduced state. Thus, eroGFP is an optical “ER stress sensor” amenable to studying ER stress in living eukaryotic cells.

Pancreatic β-cells are particularly sensitive to ER stress due to their function in synthesizing and secreting insulin. Pancreatic β-cells experience ER stress postprandially as blood glucose rises and potently stimulates proinsulin biosynthesis [9]. Furthermore, various conditions associated with obesity and type 2 diabetes cause ER stress in β-cells, which likely contributes to cell dysfunction and/or cell death [10]–[13]. Thus, monitoring ER stress by the redox-sensitive GFP would provide important information regarding how β-cells respond to ER stress induced by pharmacological as well as pathological conditions such as high levels of glucose or free fatty acids (FFAs).

In this study a redox-sensitive eroGFP was targeted to the ER of rat insulinoma cells and dispersed rat islet cells to monitor ER redox in response to various conditions that induce ER stress. We show that ER redox is perturbed by the reducing agent DTT, but not by other chemical ER stressors and conditions of physiological or pathological ER stress. Thus, ER stress in β-cells is not associated with significant ER redox (reducing) changes and suggests that pancreatic β-cell redox potential is well maintained even in the presence of severe ER stress.

## Results

### Characterization of an INS-1 cell line stably expressing an ER-localized redox-sensitive GFP and ER stress-responsive mCherry

**Figure 1:**
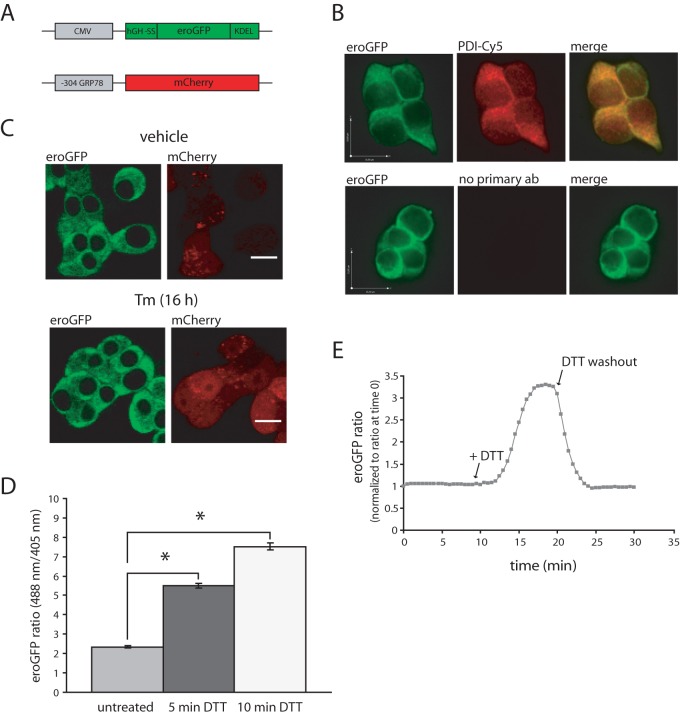
Characterization of eroGFP and mCherry expressing INS-1 (clone # 15/5) cell line. (A) Schematic diagram of constructs used to generate a double-stable INS-1 insulinoma cell line. (B) Colocalization of eroGFP with PDI by immunofluorescence. Cells were fixed and processed for immunofluorescence by using anti-PDI antibody followed by a secondary antibody labeled with Cy-5. Merging the two images demonstrates colocalization of the signals (yellow in merged image). Images were taken on a spinning disk confocal microscope. (C) Induction of mCherry upon treatment with Tm. Images were acquired on a confocal laser-scanning microscope. Scale bars: 1 µm. (D) Analysis of eroGFP emission from excitation at 488 nm/405 nm in control cells and cells treated with dithiothreitol (DTT, 5 mM) for 5 and 10 min by live cell imaging. eroGFP ratios (defined as the ratio of fluorescence from excitation at 488 nm versus 405 nm) are represented as the mean (±SEM), with a minimum of 50 cells analyzed per condition. * Denotes significance from untreated cells by Student *t*-test (*p*<0.05). (E) eroGFP ratio was monitored by confocal microscopy and image analysis. Images were taken every 30 sec. Cells were imaged for 10 min then 5 mM DTT was added. After 10 min DTT containing medium was exchanged for regular medium (DTT washout) and images were taken for another 10 min. The eroGFP ratio was calculated and normalized to the eroGFP ratio at time 0 min. Representative result from two independent experiments is shown.

To monitor ER redox in pancreatic β-cells we adapted a recently described redox-sensitive green fluorescent protein (eroGFP) used to monitor ER redox status in yeast cells [8]. We introduced a mammalian signal sequence from human growth hormone at the N-terminus and a KDEL ER retrieval motif at the C-terminus of the eroGFP to allow for expression in the ER of mammalian cells (**Fig. 1A** **, upper panel**). In addition, to monitor the transcriptional output of the UPR in response to ER stress we created a reporter construct; mCherry protein under the control of the GRP78 promoter (**Fig. 1A** **, lower panel**). The latter construct was used to first generate a stable insulinoma cell line (INS-1 GRP78mCherry # 15). This clone exhibited low basal mCherry expression and a marked induction following Tm treatment (not shown). The eroGFP construct was then used to create a double-stable insulinoma cell line (INS-1 GRP78mCherry/eroGFP # 15/5). Expression of eroGFP was confirmed by western blotting and fluorescence microscopy. ER localization of eroGFP was verified by colocalization with the ER marker protein disulfide isomerase (PDI) (**Fig. 1B**). Induction of mCherry protein expression in response to Tm treatment of the double-stable cell line confirmed that expression was sensitive to ER stress (**Fig. 1C**). However, basal and ER stress-induced expression of the mCherry probe was variable in the cell population.

The eroGFP has two fluorescence excitation maxima at approximately 400 and 490 nm and displays rapid and reversible ratiometric changes in fluorescence in response to changes in ambient redox potential. Reduction of eroGFP leads to a decrease in excitation from 400 nm, while excitation from 490 nm increases, when oxidation occurs, the situation is reversed. The ratiometric behavior of eroGFP reduces or eliminates measurement errors resulting from changes of reporter concentration, photobleaching and variable cell thickness [14], [15]. To monitor eroGFP in live cells we used confocal fluorescence microscopy and image analysis to measure the ratio of fluorescence from excitation at 488 nm versus 405 nm. Initial studies with the cell-permeable reductant dithiothreitol (DTT) demonstrated the functionality of eroGFP as an ER redox sensor in the double-stable pancreatic β-cell line. The ratio of fluorescence from the two excitation maxima increased significantly upon DTT treatment in a time-dependent fashion (**Fig. 1D**). A maximal eroGFP ratio change was observed by about 10 min of 5 mM DTT exposure. Furthermore, the effect of DTT was fully reversible. The eroGFP ratio increased to a maximum within 10 min of DTT addition and quickly reverted to baseline when DTT was washed out (**Fig. 1E**).

**Figure 2:**
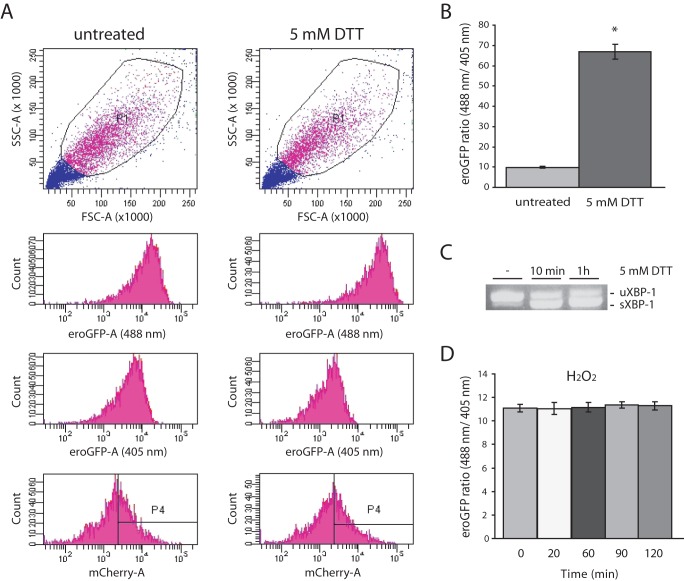
ER redox state monitored by FACS analysis in response to DTT treatment. (A) FACS plots from a representative experiment of untreated and DTT (5 mM) treated INS-1 cells (GRP78mCherry/eroGFP # 15/5). Scatter plot: forward scatter (FSC) vs. side scatter (SSC) and gated populations are represented (top left and top right panel); typical fluorescence emission of INS-1 (GRP78mCherry/eroGFP # 15/5) cells before and after addition of DTT (5 mM) for 30 min (lower left and right panels); fluorescence intensities as areas for eroGFP (488 nm), eroGFP (405 nm) and mCherry are shown. (B) eroGFP ratio (from n = 9 experiments) in cells before and after addition of 5 mM DTT (C) Ethidium bromide stained agarose gel of unspliced (uXBP-1) and spliced (sXBP-1) XBP-1 cDNA obtained by RT-PCR of total RNA of untreated and DTT treated cells. (D) eroGFP ratio (from n = 3 experiments) in cells before and after addition of 0.1 mM H_2_O_2_ at the indicated times.

Flow cytometry can also be used to monitor eroGFP and mCherry fluorescence and has the advantage of being able to monitor a larger number of live cells. INS-1 GRP78mCherry/eroGFP # 15/5 cells treated with DTT showed a reduction of eroGFP as reflected by a shift of emission from excitation at 488 nm to the right (on the x-axis of the histogram plot) towards higher fluorescence intensity and a shift of emission from excitation at 405 nm to the left towards lower fluorescence intensity (**Fig. 2A** **middle panels**). Quantitation of this change is shown in **Fig. 2B**. Induction of ER stress in cells treated with 5 mM DTT was verified by the appearance of spliced XBP-1 mRNA (**Fig. 2C**). By flow cytometry we also tested whether the eroGFP fluorescence ratio changes in response to oxidative stress i.e. to detect hyperoxidizing changes in the ER. However, treatment of cells with hydrogen peroxide did not alter the emission ratio indicating that the eroGFP probe is fully oxidized in the oxidizing environment of the ER (**Fig. 2D**) and thus unable to detect hyperoxidizing changes in the ER.

**Figure 3:**
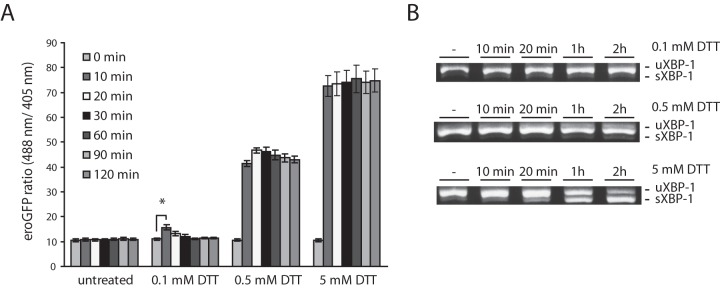
Response of cells to various concentrations of DTT over time. (A) eroGFP ratio (from n = 4 experiments) in INS-1 cells (GRP78mCherry/eroGFP # 15/5) treated or not with 0.1 mM DTT, 0.5 mM DTT and 5 mM DTT for the indicated times obtained by FACS analysis. * Denotes significance from untreated cells by Student *t*-test (*p*<0.05). (B) Ethidium bromide stained agarose gel of unspliced (uXBP-1) and spliced (sXBP-1) XBP-1 cDNA obtained by RT-PCR of total RNA of cells treated with 0.1 mM DTT, 0.5 mM DTT and 5 mM DTT at the indicated times.

We next examined the effect of lower concentrations of DTT to determine if a redox change is observed and whether the cell is able to restore redox potential in the presence of DTT. The response to addition of 5 mM DTT was rapid and resulted in fully reduced eroGFP even 2 h after treatment (**Fig. 3A**). At low concentrations of DTT (0.1 mM) however, the eroGFP ratio increased slightly, but significantly within 10 min and the redox status was re-established within 30 min. At 0.5 mM DTT the eroGFP ratio peaked at 20 min, although not to the same extent observed with a 5 mM DTT treatment. At longer times there was a trend towards a decrease in the eroGFP ratio (**Fig. 3A**). This tendency likely reflects the cells adaptation and effort to restore ER redox homeostasis. XBP-1 splicing assays were performed on cells treated with the various DTT concentrations (**Fig. 3B**). In cells exposed to 5 mM DTT spliced XBP-1 was already detected at 10 min after treatment and continued to increase over time. Cells exposed to 0.5 mM DTT showed only a small increase in spliced XBP-1 at the 1 h and 2 h time points, while spliced XBP-1 was not detected in 0.1 mM DTT treated cells.

### ER redox state is not changed by pharmacologically-induced ER Stress in INS-1 pancreatic β-cells or in dispersed rodent islet cells

The eroGFP probe was previously used to show that ER stress induced by Tm causes the redox state in the yeast ER to be more reducing [8]. To determine if different ER stressors alter redox status the double stable cell line was exposed to various conditions known to induce ER stress, including Tm, thapsigargin (Tg) and DL-homocysteine. These chemicals cause ER stress by different mechanisms, but their common effect is to interfere with ER function and thereby lead to protein misfolding and activation of the UPR.

**Figure 4:**
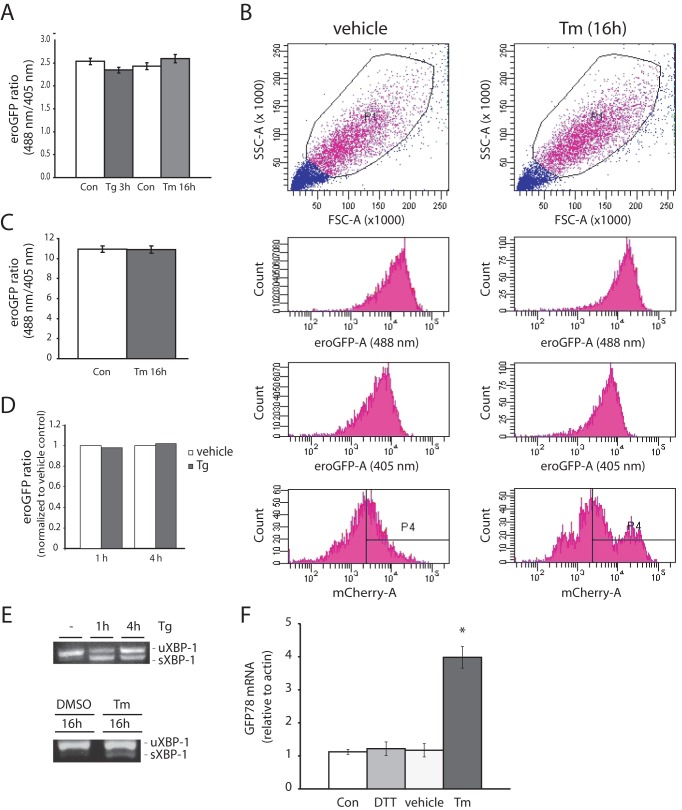
Analyses of INS-1 (GRP78mCherry/eroGFP # 15/5) cells under conditions of pharmacologically-induced ER stress. (A) eroGFP ratio of cells treated with thapsigargin (Tg) or tunicamycin (Tm) for the indicated times obtained by confocal imaging and image analysis. (B) FACS plots from a representative experiment of untreated cells or of cells treated with 2 µg/ml Tm for 16 h. Scatter plot: forward scatter (FSC) vs. side scatter (SSC) and gated population are represented (top left and top right panel); typical fluorescence emission of INS-1 (GRP78mCherry/eroGFP # 15/5) cells before and after incubation with Tm (2 µg/ml) for 16 h (lower left and right panels); fluorescence intensities as areas for eroGFP (488 nm), eroGFP (405 nm) and mCherry are shown. (C) eroGFP ratio (from n = 3 experiments) in cells treated with vehicle or treated with Tm (2 µg/ml) for 16 h obtained by FACS analysis. (D) eroGFP ratio of cells treated with Tg (1 µM) for 1 h or 4 h analyzed by FACS analysis, normalized to vehicle control. (E) Ethidium bromide stained agarose gel of unspliced (uXBP-1) and spliced (sXBP-1) XBP-1 cDNA obtained by RT-PCR of total RNA of cells after treatment with Tg (1 h and 4 h) or Tm (16 h). (F) Relative GRP78 mRNA level as determined by real-time PCR. Total RNA was isolated from GRP78mCherry/eroGFP #15/5 cells untreated, treated with DTT (5 mM) for 30 min, treated with vehicle (DMSO) for 16 h or Tm (2 µg/ml) for 16 h and real-time PCR analysis was performed to determine GRP78 mRNA expression. *Denotes significance at *p*<0.05 (ANOVA).

**Figure 5:**
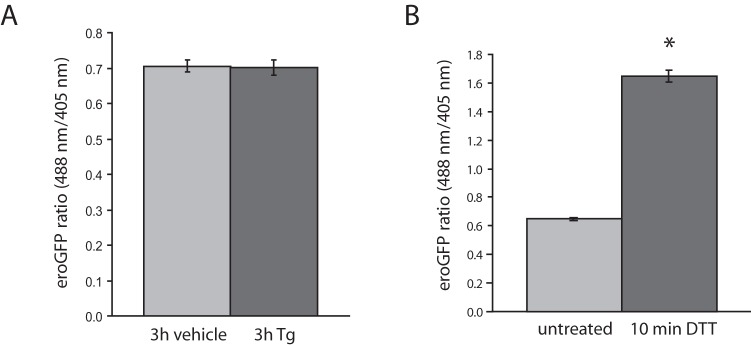
eroGFP ratio in dispersed rat islet cells. (A) eroGFP ratio in dispersed rat islet cells transduced with eroGFP adenovirus (24 h), then treated with vehicle or treated with Tg (1 µM) for 3 h. Data was obtained by confocal imaging and image analysis. (B) eroGFP ratio in rat islet cells before and after addition of 5 mM DTT for 10 min, obtained by confocal imaging and image analysis. eroGFP ratios are represented as the mean (±SEM) with a minimum of 50 cells analyzed per condition. *Denotes significance from untreated cells by Student *t*-test (*p*<0.05).

INS-1 GRP78mCherry/eroGFP # 15/5 cells treated with the ER stress-inducing agents Tm or Tg and analyzed by confocal fluorescence imaging displayed no change in eroGFP ratio (**Fig. 4A**). This result was verified using flow cytometry. The ratio of fluorescence from excitation at 488 nm versus 405 nm was not affected by Tm (**Fig. 4B, C**), although both compounds induced ER stress as monitored by XBP-1 splicing (**Fig. 4E**). An increase in mCherry fluorescence was observed in a population of cells treated with Tm, shown by a shift of emission from excitation at 532 nm to the right on the x-axis in the histogram plot (**Figure 4B** **, bottom panels**) and GRP78 mRNA levels were increased in cells treated with Tm (**Fig. 4F**). Shorter Tm treatments (5 min, 10 min and 60 min) also had no significant effect on the eroGFP ratio (results not shown). Thus, although both Tm and Tg induce ER stress the redox state of the ER is not altered. A similar result was obtained in cells from dispersed rat islets that were transduced with recombinant adenovirus expressing eroGFP. Treatment of cells with Tg had no effect on the eroGFP ratio (**Fig. 5A**), while treatment with the reducing agent DTT altered the ER redox state as expected (**Fig. 5B**).

**Figure 6:**
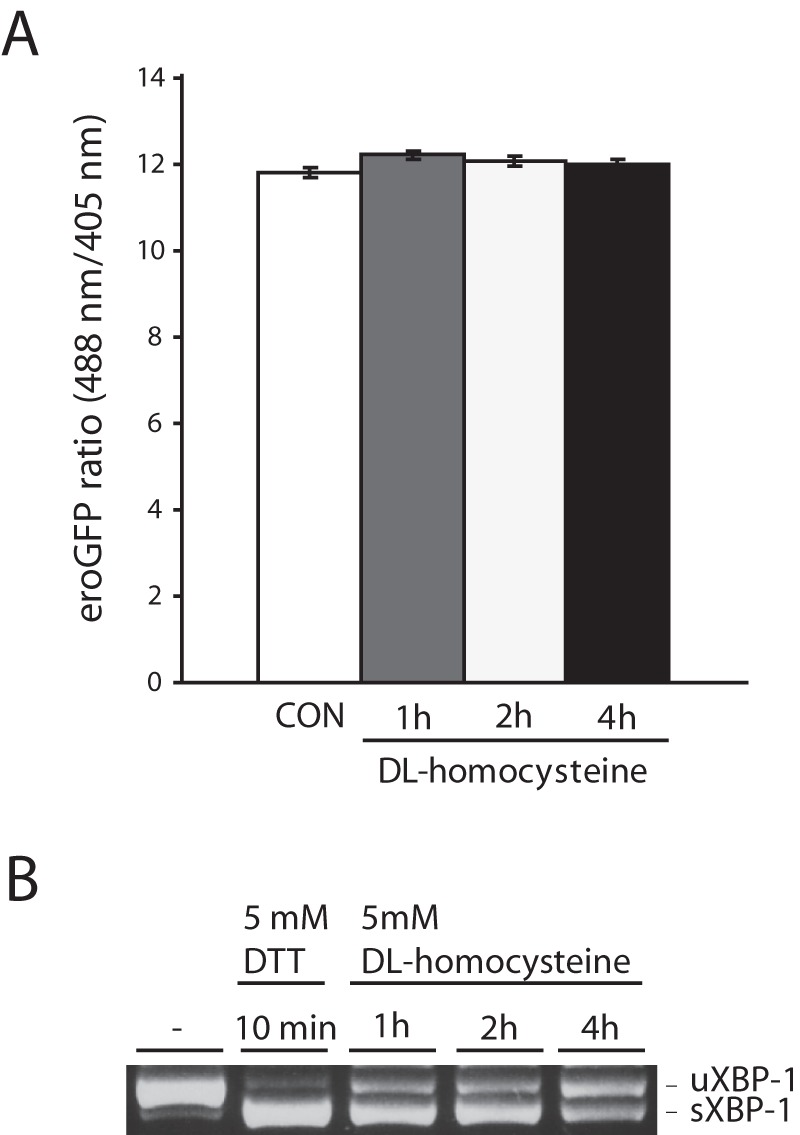
eroGFP ratio in response to ER stress induced by DL-homocysteine. (A) eroGFP ratio (from n = 3 experiments) in INS-1 cells (GRP78mCherry/eroGFP # 15/5) treated or not with 5 mM DL-homocysteine for the times indicated obtained by FACS analysis. (B) Ethidium bromide–stained agarose gel of unspliced (uXBP-1) and spliced (sXBP-1) XBP-1 cDNA obtained by RT-PCR of total RNA of cells treated or not with 5 mM DTT or 5 mM DL-homocysteine for indicated times.

Since reducing agents that induce ER stress alter the redox state of the ER we examined the effect of homocysteine a thiol-containing amino acid known to induce ER stress and alter normal disulfide bond formation [16]. Cells exposed to 5 mM DL-homocysteine showed clear evidence of ER stress (XBP-1 splicing), but the treatment had no effect on the ER redox state in INS-1 GRP78mCherry eroGFP # 15/5 cells (**Fig. 6**).

Overall, these results show that although ER stress is induced by agents such as Tm, Tg or homocysteine, this does not result in an altered (more reducing) ER environment in insulinoma cells or rodent islet cells.

### ER stress induced by obesity and diabetes-associated conditions is not associated with altered ER redox

**Figure 7:**
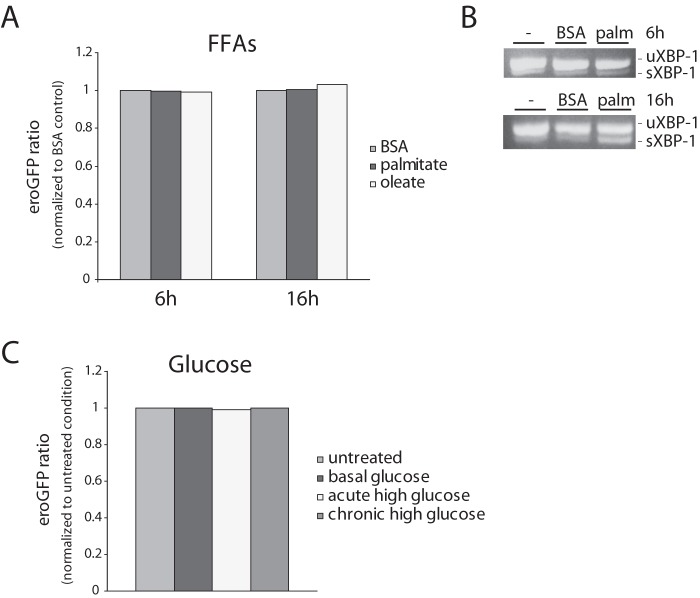
eroGFP ratio in cells treated with FFAs and high glucose. (A) eroGFP ratio of INS-1 cells (GRP78mCherry/eroGFP # 15/5) treated with palmitate and oleate for 6 h and 16 h obtained by FACS analysis normalized to BSA control. (B) Ethidium bromide–stained agarose gel of unspliced (uXBP-1) and spliced (sXBP-1) XBP-1 cDNA obtained by RT-PCR of total RNA of untreated cells, BSA treated cells and cells after treatment with palmitate (6 h and 16 h). (C) eroGFP ratio of cells under basal glucose (5 mM), acute (25 mM glucose for 2 h) and chronic high glucose (25 mM glucose for 48 h), obtained by FACS analysis. eroGFP ratios are normalized to control (untreated) cells in RPMI media (11.1 mM glucose).

Chronic exposure of pancreatic β-cells to saturated FFAs has been shown to induce ER stress [17]–[19]. We thus examined the effect of FFAs on the ER redox state. Double stable INS-1 cells were treated for 6 h or 16 h with 0.5 mM palmitate complexed with 0.5% BSA or 0.5 mM oleate complexed with 0.5% BSA in media without serum. No effect on eroGFP ratio was observed with palmitate compared to BSA-treated control cells (**Fig. 7A**). XBP-1 splicing confirmed that palmitate treatment caused ER stress (**Fig. 7B**).

Chronic exposure of β-cells to high glucose also causes ER stress and induces the UPR [20], [21]. To examine the effect of acute and chronic high glucose on ER redox double stable INS-1 cells were treated with either basal or high glucose. However, neither acute nor prolonged high glucose showed an effect on the eroGFP ratio/ER redox status (**Fig. 7C**). In summary, excess FFA or high glucose induce ER stress in pancreatic β-cells, but this is not associated with altered ER redox.

## Discussion

ER stress by definition is caused by the accumulation of unfolded, misfolded and aggregated proteins in the ER, which can be caused by a variety of factors. Accurately measuring the concentration of such aberrant proteins species, however, is not feasible. Thus, GFP based probes have been developed that monitor the luminal environment of the ER, such as calcium sensitive [22], [23] and redox sensitive probes [8], [24]. These parameters have been shown to be altered by various conditions that induce ER stress and thus ER stress can be indirectly monitored in living cells. Indeed, a recent study in yeast has shown that ER stress induced by the N-glycosylation inhibitor Tm causes a more reducing ER redox state [8]. Such a probe is useful since it allows for detection of “ER stress” in live cells and monitoring the cells' ability to restore ER homeostasis through activation of the UPR.

Here we adapted the eroGFP probe [8] to mammalian pancreatic β-cells. Unlike yeast however, chemical inducers of ER stress such as Tm or Tg, although activating the UPR, did not lead to any significant reduction in the ER redox potential. This was observed in both an insulinoma cell line and in primary cells from rodent islets. The lack of redox changes observed with Tm or Tg was not due to inability to monitor ER redox changes. The cell permeant reducing agent DTT quickly and potently reduced the normally oxidizing ER environment. Furthermore, at low concentrations of DTT, the cell was able to partially or fully restore ER redox potential depending on the ambient DTT concentration. The reason for the difference between yeast and mammalian ER redox state in response to Tm or Tg is unknown, although unlike yeast, which contains a single ER oxidase (Ero1) that maintains ER redox potential, the mammalian ER has multiple mechanisms to regulate and maintain an oxidizing environment, including Ero1 oxidases, peroxiredoxin IV, VKOR and QSOX1 [25]. Indeed, recent studies have shown that these systems contribute to maintaining an oxidizing ER environment in mammalian cells [26], [27]. Furthermore, pancreatic β-cells express high levels of the Ero1β isoform [26], [28], [29] and thus have enhanced capacity to maintain an oxidizing environment required for disulfide bond formation and folding in the lumen of the ER. An additional reason why ER reducing changes are not produced by ER stress in pancreatic β-cells could be that pancreatic β-cells are preconditioned to ER stress by expressing abundant levels of stress-responsive proteins including oxidases. Such an adapted state has been described for mammalian cells subjected to chronic low level chemical stress [30]. Chronic low level ER stress due to a high load of insulin synthesis may precondition β-cells (i.e. the UPR is active to a certain extent at all times), allowing β-cells to better deal with additional stress that may affect the ER environment [31]. This form of preconditioning could contribute to the differences in ER redox homeostasis in response to ER stress in β-cells compared to yeast.

ER redox was also unaltered in insulinoma cells exposed to chronic palmitate or homocysteine treatment, both of which induced ER stress as detected by spliced XBP-1 mRNA levels. Some studies have shown that palmitate treatment causes ER calcium depletion that contributes to causing ER stress [32]. Homocysteine however is a redox-active amino acid and has been suggested to alter the cellular redox state, thereby causing ER stress [16]. The toxic effects of homocysteine have been frequently attributed to direct or indirect perturbation of redox homeostasis. Thus, it was unexpected that homocysteine did not change the redox potential in pancreatic β-cells. However, homocysteine induces varied responses that are cell type specific and cells have a wide range of sensitivity to homocysteine [16]. Furthermore, homocysteine also causes depletion of ER calcium, which can contribute to causing ER stress independent of redox changes [16].

Yeast cells lacking a normal UPR (deletions of either IRE1 or Hac1 genes) when treated with Tm exhibit a greater eroGFP ratio change (more reducing ER) in the UPR mutants than in wild-type cells [8]. The UPR induces enzymes that mediate ER protein oxidation such as Ero1 and PDI and without their induction the cell is not able to efficiently counteract ER reducing changes. Thus, it is possible that we did not detect a more reducing ER in pancreatic β-cells in response to ER stress due to activation of the UPR, which ultimately increases oxidative ER capacity.

Importantly, the redox sensitive GFP probe we used in this study is sufficient to monitor changes in the reducing direction. However, due to the probes reduction potential, it is essentially fully oxidized in the oxidizing environment of the ER and thus is not useful for detecting hyperoxidizing ER conditions [24]. Thus, it is possible that the various ER stressors tested in this study could alter ER redox potential by causing a more oxidized ER redox environment. Indeed, the CHOP transcription factor which is induced by the UPR in response to ER stress activates ER oxidases including ERO1α perhaps resulting in a hyperoxidizing ER [33]. In fact, CHOP deletion protects cells from ER stress in part by causing a more reducing ER environment. The use of newer probes such as the GFP-iL [24], which has a redox balance that allows monitoring of redox changes in both a more reducing and a more oxidizing ER environment will be useful in assessing whether different ER stressors result in hyperoxidation of the ER.

In conclusion, we found that ER redox state does not become more reducing in pancreatic β-cells in the presence of ER stress induced by a variety of conditions. Consequently, ER redox state, at least with the eroGFP probe used in this study, is not a good indicator for ER stress in pancreatic β-cells. Future studies are required to develop a system able to reveal the extent of ER stress caused by physiological and pathophysiological conditions and to examine restoration of ER homeostasis in living cells. These investigations will further our understanding of the ER stress response in pancreatic β-cells.

## Materials and Methods

### Ethics Statement

All protocols using animals in this study were approved by the Animal Resources Centre at the University Health Network, Toronto.

### Antibodies

Mouse monoclonal anti-GFP (Clontech, 632381, 1∶1000), mouse monoclonal anti-DsRed (Clontech, 632393 1∶400), mouse monoclonal anti-KDEL (StressGen, SPA-827, 1∶1000), rabbit polyclonal protein disulfide isomerase (PDI), (StressGen; SPA-890, 1∶250), secondary Cy5-conjugated anti-rabbit antibody (Jackson Immuno Research, 1∶250).

### Reagents

Tunicamycin (Tm), Thapsigargin (Tg) and hydrogene peroxide (H_2_O_2_) were from Sigma-Aldrich, Dithiothreitol (DTT) was from Fisher Biotech, DL-homocysteine was from Santa Cruz Biotechnology. Palmitate and oleate were from Sigma (P-0500 and O-7501).

### Generation of a plasmid containing mCherry under the control of the stress inducible −304 GRP78 promoter

To create the pGRP78mCherry plasmid the vector pmCherry-N1 (Clontech) was used as the backbone. The CMV promoter sequence was excised from pmCherry-N1 by using AseI and NheI restriction sites, the vector was blunt ended with T4 DNA polymerase and then religated to give pmCherryN1 Blunt. The pGL2 plasmid containing the human -304 GRP78 promoter sequence was digested with KpnI and HindIII. The KpnI-HindIII fragment containing the −304 GRP78 promoter sequence was subcloned into the restriction sites KpnI and HindIII of pcDNA3.1+ (Invitrogen). The XhoI-HindIII fragment of this construct was then cloned into the XhoI/HindIII sites of pmCherryN1 Blunt vector to generate pGRP78mCherry.

### Generation of a plasmid expressing an ER localized redox-sensitive GFP (eroGFP)

The hGHSS was amplified from pC4S1-F(M)4-HA by PCR using the Platinum Taq DNA Polymerase High Fidelity from Invitrogen and the following primers containing a 5′ NheI restriction site and a 3′ SalI restriction site (forward primer 5′-TTAAGCTAGCATGGCTACAGGCTCCCGG-3′ and reverse primer 5′-TATAGTCGACTTGGCACTGCCCTCTTGAAGC-3′). PCR was performed for 1 cycle of 94°C for 1 min followed by 35 cycles of 15 s at 94°C, 15 s at 55°C, and 25 s at 68°C and 1 cycle of 68°C for 10 min. EGFP was removed from pEGFP-N1 (Clontech), used as the backbone to create phGHSS-eroGFP-KDEL by using AgeI and NotI restriction sites and blund ended using T4 DNA polymerase. The vector was religated and the NheI-SalI fragment containing the hGHSS sequence was introduced into NheI and SalI restriction sites. The eroGFP sequence was excised from the yeast plasmid pPM28 (Addgene plasmid 20131) [8] using BamHI and XbaI restriction sites and was subcloned into pCRII-Topo (Invitrogen) vector. The KpnI-ApaI fragment of the so generated plasmid was then cloned in frame into the blund ended plasmid containing the hGHSS. The HDEL ER retrieval signal was converted into KDEL by site directed mutagenesis using the Stratagene's QuikChange II XL site-directed mutagenesis kit following the manufacturer's instructions to give the plasmid phGHSS-eroGFP-KDEL. The construct's sequence was confirmed by DNA sequencing with appropriate primers.

### Cell culture and generation of a double stable INS-1 based β-cell line expressing mCherry and eroGFP

INS-1 pancreatic β-cells (obtained from Dr. Claes Wollheim, University of Geneva) [34] were maintained in RPMI 1640 (11.1 mM glucose, 1 mM sodium pyruvate, 10 mM HEPES) supplemented with 10% fetal bovine serum, 2 mM L-glutamine, and 55 µM β-mercaptoethanol and a standard tissue culture penicillin-streptomycin mixture (100 units/ml penicillin and 100 μg/ml streptomycin) in a humidified incubator under 5% CO_2_ at 37°C. Initially a stable INS-1-GRP78mCherry clone was generated by transfecting INS-1 cells with the plasmid pGRP78mCherry. Cells were cultivated under selective pressure with 400 µg/ml geneticin (Invitrogen) to select stable clones. Several geneticin resistant clones were isolated and examined for mCherry expression by Western Blotting and fluorescence microscopy and for their ability to respond to Tm treatment with an increase in the level of mCherry protein expression and mCherry fluorescence. A clone with a low basal expression of mCherry and with the strongest Tm-dependent induction of mCherry (INS-1-GRP78mCherry #15) was selected and used for the generation of the double stable cell line. Double-stable INS-1-GRP78mCherry-eroGFP expressing cells were generated by co-transfection of phGHSS-eroGFP-KDEL with a hygromycin resistance plasmid into INS-1-GRP78mCherry #15 cells and screening for the selection marker by addition of 400 µg/ml geneticin (Invitrogen) and 50 µg/ml hygromycin B (Invitrogen). Individual clones were isolated and tested for eroGFP and mCherry expression by Western Blotting and fluorescence. DTT treatment (5 mM) followed by confocal fluorescence microscopy was used to confirm the eroGFP's redox properties. A positive clone INS-1-GRP78mCherry-eroGFP #15/5 was identified. The generated single stable and double stable cell lines were maintained in RPMI 1640 (11.1 mM glucose, 1 mM sodium pyruvate, 10 mM HEPES) supplemented with 10% fetal bovine serum, 2 mM L-glutamine, 55 µM β-mercaptoethanol with antibiotics (100 units/ml penicillin and 100 µg/ml streptomycin) and selection drugs (50 µg/ml hygromycin and/or 400 µg/ml geneticin).

### Recombinant adenovirus production

Adenovirus expressing eroGFP was generated using the AdEasy XL Adenoviral System (Stratagene) and was prepared as described previously [18]. Briefly, the eroGFP sequence was amplified from the phGHSS-eroGFP-KDEL plasmid by PCR with Platinum Taq (Invitrogen) to attach a 5′ XhoI and a 3′ HindIII restriction site. The following primers were used to incorporate the restriction sites: forward primer 5′-TATACTCGAGATGGCTACAGGCTCCCGG-3′ and reverse primer 5′-TATTAAGCTTTTACAATTCGTCCTTCTTGTACAATTCG-3′. PCR amplification was performed in the following sequence: 1 cycle of 94°C for 1 min followed by 30 cycles of 30 s at 94°C, 30 s at 54°C, and 1 mins at 68°C and 1 cycle of 68°C for 10 min. The *Xho*I/HindIII fragment representing the eroGFP was subcloned into the pShuttleCMV plasmid backbone. Plasmid DNA isolated from positive colonies was linearized with *Pme*I and electroporated into BJ5183-AD-1 bacterial stocks for recombination into the pADEasy plasmid backbone. Plasmids from positive recombinants of eroGFP were transformed into *E. coli* XL-10 Gold cells for large-scale DNA preparation. For primary virus production, linearized plasmid (PacI digested) was used to transiently transfect AD-293 cells. Adenovirus-producing AD293 cells were harvested and lysed by four rounds of freeze/thaw lysis. The viral particles were subjected to one round of amplification. Successful construction of the recombinant virus was verified by Western Blotting and confocal fluorescence imaging and image analysis. Viral stocks were then used for further viral amplification in AD293 cells. Amplified recombinant adenovirus was purified using the Vivapure AdenoPACK 100 kit (Vivascience). The purified virus was resuspended in buffer (20 mM Tris/HCl, 25 mM NaCl, 2.5% glycerol, pH 8.0), and aliquots were stored at −80°C. The viral titer was determined by measuring the absorbance at 260 nm. The titer was 1.3×10^12^ PFU/ml.

### Rat islet isolation, dispersion of islets and adenoviral transduction

Rat islets were isolated from male Wistar rats weighing ∼250–300 g by using a collagenase digestion followed by separation using a density gradient as previously described [35]. Briefly, animals were housed and anesthetized by intraperitoneal injection of a mixture of rompum and ketalean following protocols approved by the Animal Resources Centre at the University Health Network. Under anesthesia, a laparotomy was performed, and the pancreas was exposed. 15 ml collagenase solution (Sigma) was injected into the pancreas via the common bile duct. The pancreas was removed and incubated in a water bath for 20 min at 37°C with gentle tumbling. The islets were separated by a density gradient (Histopaque-1077; Sigma) and centrifuged at 2700 rpm for 23 min at 4°C. The islets floating on the interphase of the gradient were collected and sedimented. After washing, islets were handpicked and transferred into cell culture dishes. ∼350 islets were extracted per pancreas and transferred into RPMI media containing 10% fetal bovine serum, 11 mM glucose, 100 U/ml penicillin and 100 µg/ml streptomycin, for recovery over night. Following recovery, ∼200 islets per condition, were picked by hand, transferred into tubes and washed with PBS pH 7.4. In order to obtain dispersed β-cells islets were treated with PBS supplemented with 2 mM ethylene glycol tetra-acetic acid (EGTA, Fisher Biotech) for 9 min followed by digestion with Accutase (Millipore) for 5 min at 37°C (gentle shaking, 2–3 x). Remaining intact islets were disrupted mechanically by pipetting. Cells were seeded and cultured for 48 h at 37°C in a humidified 5% CO2 incubator in RPMI medium containing 10% of fetal bovine serum, but without antibiotics. Then cells were infected with recombinant adenovirus expressing eroGFP and cultured at 37°C for another 24 h before being exposed to DTT (5 mM, 10 min) and Tg (1 µM, 3 h). After treatment fluorescent cells were analyzed using confocal fluorescence microscopy and image analysis and their eroGFP ratio was determined as described below.

### Cell lysis and western blot analysis

Cells were washed in PBS and lysed in ice-cold lysis buffer (1% Triton X-100, 100 mM KCl, 20 mM HEPES, 2 mM EDTA, pH 7.4, supplemented with 1 mM PMSF and Roche protease inhibitor tablet) for 30 min on ice. Cell lysates were centrifuged at 13,000 rpm for 10 min at 4°C and the protein concentration in the supernatant was determined using a BCA protein assay (Pierce). Equal amounts of total protein were resolved by denaturing sodium dodecyl sulphate polyacrylamide gel electrophoresis (SDS-PAGE) on 10% gels and immunoblotted as described previously [20]. The following primary antibodies were used: monoclonal (mouse) anti-GFP (Clontech, 632381), monoclonal (mouse) anti-DsRED (Clontech, 632393), and monoclonal (mouse) anti-KDEL (StressGen, SPA-827).

### Fluorescence microscopy

For screening purposes the various clones were seeded on glass coverslips in 24-well dishes treated or not with Tm (2 µg/ml), washed with PBS and fixed with 3% paraformaldehyde in PBS for 20 minutes, washed with PBS and mounted on glass coverslips using Fluoromount G (EM Sciences). Fluorescence of mCherry and eroGFP were visualized with an Olympus inverted fluorescence microscope (IX71, Tokyo, Japan) equipped with an Olympus PlanApo 60×/1.42 NA oil objective and images were captured using Q-Capture Pro software (Q Imaging, Surrey, Canada).

### Immunofluoresence, image acquisition and processing

For colocalization studies INS-1-GRP78mCherry-eroGFP #15/5 cells were seeded on glass coverslips in 24-well dishes. After an overnight incubation, the cells were washed with PBS and fixed with 3% paraformaldehyde in PBS for 20 min. The cells were further processed for immunofluorescence microscopy as reported previously [17] and immunostained with anti-PDI (rabbit) antibody at a 1∶250 dilution and Cy5-conjugated secondary antibody. Negative controls for antibody staining were prepared as described above, except that the primary antibody was omitted. Colocalization of eroGFP with the ER marker was confirmed by spinning disk confocal fluorescence microscopy. Images of fixed cells were acquired using a Zeiss Axiovert 200 inverted microscope equipped with a Hamamatsu Orca AG CCD camera and spinning disk confocal scan head. For fluorescence excitation diode-pumped solid state laser lines were used, excitation line 491 nm for eroGFP excitation and line 655 nm for excitation of Cy5. Fluorescence signals were collected with a 63×, NA 1.3 water immersion objective lens (Zeiss). Images were captured using Volocity 4 acquisition software.

### Confocal fluorescence microscopy of live cells and ImageJ processing

Image acquisition for the calculation of the eroGFP ratio under various conditions was performed on a FluoView1000 confocal microscope (Olympus) with a built in incubator with temperature and CO_2_ control using an Olympus PlanApo 60×/1.4 NA oil objective. The microscope stage was maintained at 37°C and 5% CO_2_. Wavelengths (ex/em) of 405 nm/510–540 nm for eroGFP (band pass filter), 488 nm/510–540 nm (band pass filter) for eroGFP and 543 nm/612 nm for mCherry were applied. Fluorescence of eroGFP (488 nm) was excited with a 488 nm multiline Argon laser and eroGFP (405 nm) was excited with a 405 nm diode laser, while mCherry fluorescence was excited with a 543 nm Helium–Neon laser. Cells were seeded into 4-chambered Lab-Tek II chamber slides (Nalgene Nunc International, Thermo Scientific) at 400 000 cells per chamber in RPMI 1640 media and incubated overnight. For initial studies of eroGFP behaviour live cells maintained in RPMI 1640 media were stimulated with 5 mM DTT and changes in eroGFP fluorescence were followed by confocal microscopy at 37°C and 5% CO_2_ for 10 min. The chemical agents Tg (1 µM, 3 h) and Tm (2 µg/ml, 16 h) were used as positive controls for induction of ER stress. Randomly selected fields from each slide were imaged, and channels were scanned sequentially to maximize separation. Images of one focal plane of the cells were taken and all images in a given experiment were captured and analyzed with the same exposure time and conditions (laser intensity). Measurements were made at the given time points (and samples kept at 37°C in between measurements.) Documented images were processed for eroGFP ratio using ImageJ software. Image data sets were analysed using ImageJ 1.42q (Rasband, W.S., ImageJ, U. S. National Institutes of Health, Bethesda, Maryland, USA, http://rsb.info.nih.gov/ij/, 1997–2008). In brief, freehand line selection was drawn along the cell's edge and the integrated intensity of eroGFP (488 nm excitation) and eroGFP (405 nm excitation) in arbitrary units in cells was determined. Ratio of eroGFP was calculated using Microsoft Excel. A minimum of 50 cells for all experimental conditions was quantified and the mean eroGFP ratio is reported.

### Flow cytometry analysis

To assess the effect of various ER stressors on the eroGFP ratio and mCherry expression INS1 GRP78mCherry eroGFP #15/5 cells were incubated with DTT, Tm, Tg, DL-homocysteine, FFAs (palmitate and oleate) and high glucose. For flow cytometric quantification of mCherry and eroGFP fluorescence INS-1-GRP78mCherry-eroGFP #15/5 cells were grown in 6-well plates. Cells (1000000 cells per well), were seeded 24 h to 48 h before treatment followed by incubation with 1 µM Tg (1 h and 4 h), 2 µg/ml Tm (16 h), 5 mM DL-homocysteine (1 h, 2 h and 4 h) and high glucose (25 mM) for 2 h (acute) and 48 h (chronic) or stimulation of cells with 0.5 mM FFA/0.5% BSA for 6 h and 16 h. DTT treatments were performed at final concentrations of 0.1 mM, 0.5 mM and 5 mM. Cells were treated with H_2_O_2_ at a final concentration of 0.1 mM. As a positive control in all the experiments cells treated with 5 mM DTT for 10 min were included. Untreated INS-1 cells were used as a negative control. Following treatments, live cells were washed with warm PBS and detached with 10 mM EDTA in PBS. Cells were pelleted by a low speed centrifugation step and dissolved in RPMI media without phenol red and serum, supplemented with 0.1 mM EDTA to obtain single-cell suspensions. Tm, Tg, DTT and DL-homocysteine were added, respectively. Cells incubated with basal or high glucose were dissolved in KRBH buffer (128.8 mM NaCl, 4.8 mM KCl, 1.2 mM KH_2_PO_4_, 1.2 mM MgSO_4_, 2.5 mM CaCl_2_, 5 mM NaHCO_3_, 10 mM Hepes, 0.1% bovine serum albumin, pH 7.4) supplemented with 5 mM glucose (basal) or 25 mM glucose (high glucose) and 0.1 mM EDTA. For the DTT and H_2_O_2_ experiment over a time of 2 h cells were first prepared as described above and afterwards the single cell suspension were treated with either DTT or H_2_O_2_ and the effect on the eroGFP ratio monitored. Flow cytometric analyses were conducted on a FACS-LSRII (Becton Dickinson) flow cytometer. Cells were excited using laser lines at 405 nm (eroGFP 405 nm), laser line 488 nm (eroGFP 488 nm) and laser line 532 nm (mCherry). The emission filter setup was the following: long pass filter 505 nm and band pass filter 510/21 nm for eroGFP (405 nm), long pass filter 505 nm and band pass filter 510/20 for eroGFP (488 nm) and long pass filter 600 nm and band pass filter 610/20 nm for mCherry. Data analysis and eroGFP calculation was performed using the BD FACSDiva software version 6.0. The eroGFP ratios for each treatment were normalized to INS-1-GRP78mCherry-eroGFP #15/5 control condition were indicated.

### FFA preparation and cell treatment

FFA solutions were prepared as described previously [36]. Briefly, 100 mM palmitate and 100 mM oleate stocks were prepared in 0.1 M NaOH at 70°C and filtered. Five percent (wt/vol) FFA-free BSA (Sigma no. A-6003) solution was prepared in double-distilled H_2_O and filtered. A 5 mM FFA/5% BSA solution was prepared by complexing an appropriate amount of FFA to 5% BSA at 60°C. The solution was then cooled to room temperature and diluted 1∶10 in RPMI 1640 without FBS to obtain a final concentration of 0.5 mM FFA/0.5% BSA.

### XBP-1 splicing assay

Total RNA was isolated using TRIzol reagent (Invitrogen) and was further purified with Qiagen RNeasy minicolumns according to the manufacturer's instructions. Rat XBP-1 cDNA was amplified by RT-PCR (Qiagen OneStep RT-PCR kit) using primers that flank the intron excised by IRE1 exonuclease activity as described previously [20].

### Real time PCR analysis

For real-time PCR analysis, total RNA was isolated from cells treated as described in the figure legend. Reverse transcription was performed with a high-capacity cDNA reverse transcription kit (Applied Biosystems). The resulting cDNA was used for real-time PCR analysis using the TaqMan gene expression system (Applied Biosystems) as described in [20]. The following TaqMan MGB probes were used: rat GRP78 (Rn01435771_g1) and rat β-actin (rat ACTB, 4352931E).

### Data analysis

Results are presented as mean +/− SE. Statistical significance between two experimental conditions was analyzed by Student's two-sample t tests assuming equal variances and between several groups using one-way ANOVA, where P≤0.05 was considered statistically significant.

## References

[1] Ellgaard L, Helenius A (2003) Quality control in the endoplasmic reticulum. Nature reviews Molecular cell biology 4: 181–191. 12612637 10.1038/nrm1052

[2] Papp S, Dziak E, Michalak M, Opas M (2003) Is all of the endoplasmic reticulum created equal? The effects of the heterogeneous distribution of endoplasmic reticulum Ca2+-handling proteins. The Journal of cell biology 160: 475–479. 12591911 10.1083/jcb.200207136 PMC2173736

[3] Sevier CS, Kaiser CA (2002) Formation and transfer of disulphide bonds in living cells. Nature reviews Molecular cell biology 3: 836–847. 12415301 10.1038/nrm954

[4] Boyce M, Yuan J (2006) Cellular response to endoplasmic reticulum stress: a matter of life or death. Cell death and differentiation 13: 363–373. 16397583 10.1038/sj.cdd.4401817

[5] Volchuk A, Ron D (2010) The endoplasmic reticulum stress response in the pancreatic β-cell. Diabetes, obesity & metabolism 12 Suppl 2: 48–57. 10.1111/j.1463-1326.2010.01271.x 21029300

[6] Lai E, Teodoro T, Volchuk A (2007) Endoplasmic reticulum stress: signaling the unfolded protein response. Physiology (Bethesda, Md) 22: 193–201. 10.1152/physiol.00050.2006 17557940

[7] Ron D, Walter P (2007) Signal integration in the endoplasmic reticulum unfolded protein response. Nature reviews Molecular cell biology 8: 519–529. 17565364 10.1038/nrm2199

[8] Merksamer PI, Trusina A, Papa FR (2008) Real-time redox measurements during endoplasmic reticulum stress reveal interlinked protein folding functions. Cell 135: 933–947. 19026441 10.1016/j.cell.2008.10.011 PMC2739138

[9] Kim M-K, Kim H-S, Lee I-K, Park K-G (2012) Endoplasmic reticulum stress and insulin biosynthesis: a review. Experimental diabetes research 2012: 509437. 22474424 10.1155/2012/509437 PMC3303544

[10] Fonseca SG, Burcin M, Gromada J, Urano F (2009) Endoplasmic reticulum stress in beta-cells and development of diabetes. Current opinion in pharmacology 9: 763–770. 19665428 10.1016/j.coph.2009.07.003 PMC2787771

[11] Hotamisligil GS (2010) Endoplasmic reticulum stress and the inflammatory basis of metabolic disease. Cell 140: 900–917. 20303879 10.1016/j.cell.2010.02.034 PMC2887297

[12] Rieusset J (2011) Mitochondria and endoplasmic reticulum: mitochondria-endoplasmic reticulum interplay in type 2 diabetes pathophysiology. The international journal of biochemistry & cell biology 43: 1257–1262. 21605696 10.1016/j.biocel.2011.05.006

[13] Cnop M, Foufelle F, Velloso LA (2012) Endoplasmic reticulum stress, obesity and diabetes. Trends in molecular medicine 18(1): 59–68. 21889406 10.1016/j.molmed.2011.07.010

[14] Hanson GT, Aggeler R, Oglesbee D, Cannon M, Capaldi RA, et al (2004) Investigating mitochondrial redox potential with redox-sensitive green fluorescent protein indicators. The Journal of biological chemistry 279: 13044–13053. 14722062 10.1074/jbc.M312846200

[15] Dooley CT, Dore TM, Hanson GT, Jackson WC, Remington SJ, et al (2004) Imaging dynamic redox changes in mammalian cells with green fluorescent protein indicators. The Journal of biological chemistry 279: 22284–22293. 14985369 10.1074/jbc.M312847200

[16] Zou C-G, Banerjee R (2005) Homocysteine and redox signaling. Antioxid Redox Signal 7: 547–559. 15890000 10.1089/ars.2005.7.547

[17] Karaskov E, Scott C, Zhang L, Teodoro T, Ravazzola M, et al (2006) Chronic palmitate but not oleate exposure induces endoplasmic reticulum stress, which may contribute to INS-1 pancreatic beta-cell apoptosis. Endocrinology 147: 3398–3407. 16601139 10.1210/en.2005-1494

[18] Lai E, Bikopoulos G, Wheeler MB, Rozakis-Adcock M, Volchuk A (2008) Differential activation of ER stress and apoptosis in response to chronically elevated free fatty acids in pancreatic beta-cells. American journal of physiology Endocrinology and metabolism 294: E540–50. 18198352 10.1152/ajpendo.00478.2007

[19] Cnop M, Ladriere L, Hekerman P, Ortis F, Cardozo AK, et al (2007) Selective inhibition of eukaryotic translation initiation factor 2 alpha dephosphorylation potentiates fatty acid-induced endoplasmic reticulum stress and causes pancreatic beta-cell dysfunction and apoptosis. The Journal of biological chemistry 282: 3989–3997. 17158450 10.1074/jbc.M607627200

[20] Zhang L, Lai E, Teodoro T, Volchuk A (2009) GRP78, but Not Protein-disulfide Isomerase, Partially Reverses Hyperglycemia-induced Inhibition of Insulin Synthesis and Secretion in Pancreatic {beta}-Cells. The Journal of biological chemistry 284: 5289–5298. 19103594 10.1074/jbc.M805477200

[21] Elouil H, Bensellam M, Guiot Y, Vander Mierde D, Pascal SM, et al (2007) Acute nutrient regulation of the unfolded protein response and integrated stress response in cultured rat pancreatic islets. Diabetologia 50: 1442–1452. 17497122 10.1007/s00125-007-0674-4

[22] Demaurex N, Frieden M (2003) Measurements of the free luminal ER Ca2+ concentration with targeted “cameleon” fluorescent proteins. Cell Calcium 34: 109–119. 12810053 10.1016/s0143-4160(03)00081-2

[23] Solovyova N, Verkhratsky A (2002) Monitoring of free calcium in the neuronal endoplasmic reticulum: an overview of modern approaches. Journal of neuroscience methods 122: 1–12. 12535760 10.1016/s0165-0270(02)00300-x

[24] van Lith M, Tiwari S, Pediani J, Milligan G, Bulleid NJ (2011) Real-time monitoring of redox changes in the mammalian endoplasmic reticulum. Journal of cell science 124: 2349–2356. 21693587 10.1242/jcs.085530 PMC3124370

[25] Margittai E, Bánhegyi G (2010) Oxidative folding in the endoplasmic reticulum: towards a multiple oxidant hypothesis? FEBS letters 584: 2995–2998. 20621831 10.1016/j.febslet.2010.05.055

[26] Zito E, Chin K-T, Blais J, Harding HP, Ron D (2010) ERO1-beta, a pancreas-specific disulfide oxidase, promotes insulin biogenesis and glucose homeostasis. The Journal of cell biology 188: 821–832. 20308425 10.1083/jcb.200911086 PMC2845084

[27] Rutkevich LA, Williams DB (2012) Vitamin K epoxide reductase contributes to protein disulfide formation and redox homeostasis within the endoplasmic reticulum. Molecular biology of the cell 23: 2017–2027. 22496424 10.1091/mbc.E12-02-0102 PMC3364168

[28] Dias-Gunasekara S, Gubbens J, van Lith M, Dunne C, Williams JA, et al (2005) Tissue-specific expression and dimerization of the endoplasmic reticulum oxidoreductase Ero1beta. The Journal of biological chemistry 280: 33066–33075. 16012172 10.1074/jbc.M505023200

[29] Pagani M, Fabbri M, Benedetti C, Fassio A, Pilati S, et al (2000) Endoplasmic reticulum oxidoreductin 1-lbeta (ERO1-Lbeta), a human gene induced in the course of the unfolded protein response. The Journal of biological chemistry 275: 23685–23692. 10818100 10.1074/jbc.M003061200

[30] Rutkowski DT, Arnold SM, Miller CN, Wu J, Li J, et al (2006) Adaptation to ER stress is mediated by differential stabilities of pro-survival and pro-apoptotic mRNAs and proteins. PLoS biology 4: e374. 17090218 10.1371/journal.pbio.0040374 PMC1634883

[31] Vellanki RN, Zhang L, Guney MA, Rocheleau JV, Gannon M, et al (2010) OASIS/CREB3L1 induces expression of genes involved in extracellular matrix production but not classical endoplasmic reticulum stress response genes in pancreatic beta-cells. Endocrinology 151: 4146–4157. 20668028 10.1210/en.2010-0137 PMC2940493

[32] Jeffrey KD, Alejandro EU, Luciani DS, Kalynyak TB, Hu X, et al (2008) Carboxypeptidase E mediates palmitate-induced beta-cell ER stress and apoptosis. Proceedings of the National Academy of Sciences of the United States of America 105: 8452–8457. 18550819 10.1073/pnas.0711232105 PMC2448857

[33] Marciniak SJ, Yun CY, Oyadomari S, Novoa I, Zhang Y, et al (2004) CHOP induces death by promoting protein synthesis and oxidation in the stressed endoplasmic reticulum. Genes & development 18: 3066–3077. 15601821 10.1101/gad.1250704 PMC535917

[34] Asfari M, Janjic D, Meda P, Li G, Halban PA, et al (1992) Establishment of 2-mercaptoethanol-dependent differentiated insulin-secreting cell lines. Endocrinology 130: 167–178. 1370150 10.1210/endo.130.1.1370150

[35] Macdonald PE, Ha XF, Wang J, Smukler SR, Sun AM, et al (2001) Members of the Kv1 and Kv2 Voltage-Dependent K Channel Families Regulate Insulin Secretion. Physiology 15: 1423–1435. 10.1210/mend.15.8.0685 11463864

[36] Cousin SP, Hugl SR, Wrede CE, Kajio H, Myers MG Jr, et al (2001) Free fatty acid-induced inhibition of glucose and insulin-like growth factor I-induced deoxyribonucleic acid synthesis in the pancreatic beta-cell line INS-1. Endocrinology 142: 229–240. 11145586 10.1210/endo.142.1.7863

